# Supplementary material for: The Ancient History of Peptidyl Transferase Center Formation as Told by Conservation and Information Analyses
Source: Life (Basel). 2020 Aug 5;10(8):134. doi: 10.3390/life10080134 (PMC7459865; doi:10.3390/life10080134)
Supplement: Supplementary file 1 [file life-10-00134-s001.zip › life-875402-supplementary/life-875402-Supplementary Materials.docx]

The Ancient History of Peptidyl Transferase Center Formation as Told by Conservation and Information Analyses

Francisco Prosdocimi ^1,2,^*, Gabriel S. Zamudio ^2^, Miryam Palacios-Pérez ^2^, Sávio Torres de Farias ^3^ and Marco V. José ^2,^*

^1^ Laboratório de Biologia Teórica e de Sistemas, Instituto de Bioquímica Médica Leopoldo de Meis, Universidade Federal do Rio de Janeiro, Rio de Janeiro 21.941-902, Brazil

^2^ Theoretical Biology Group, Instituto de Investigaciones Biomédicas, Universidad Nacional Autónoma de México, Ciudad Universitaria, CDMX 04510, Mexico; gazaso92@gmail.com (G.S.Z.); mir.pape@iibiomedicas.unam.mx (M.P.-P.)

^3^ Laboratório de Genética Evolutiva Paulo Leminsk, Departamento de Biologia Molecular, Universidade Federal da Paraíba, João Pessoa, Paraíba 58051-900, Brazil; stfarias@yahoo.com.br

***** Correspondence: prosdocimi@bioqmed.ufrj.br (F.P.); marcojose@biomedicas.unam.mx (M.V.J.)


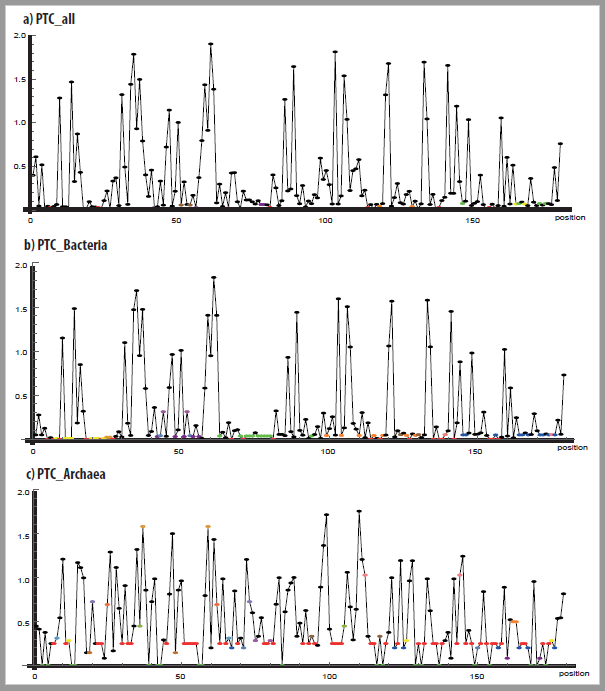


**Figure S1.** Variation of information (bits) along the sequence of the PTC: (a) PTC-all (b) PTC-Bacteria, and (c) PTC-Archaea.


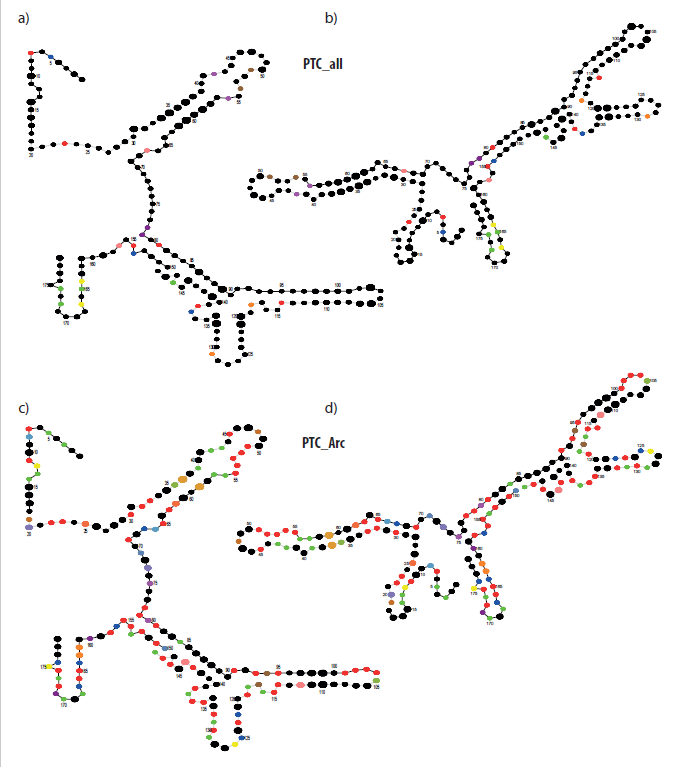


**Figure S2.** Relationship between proto-tRNA mapping and information clusters for (a) PTC-all and (b) PTC-Arc.


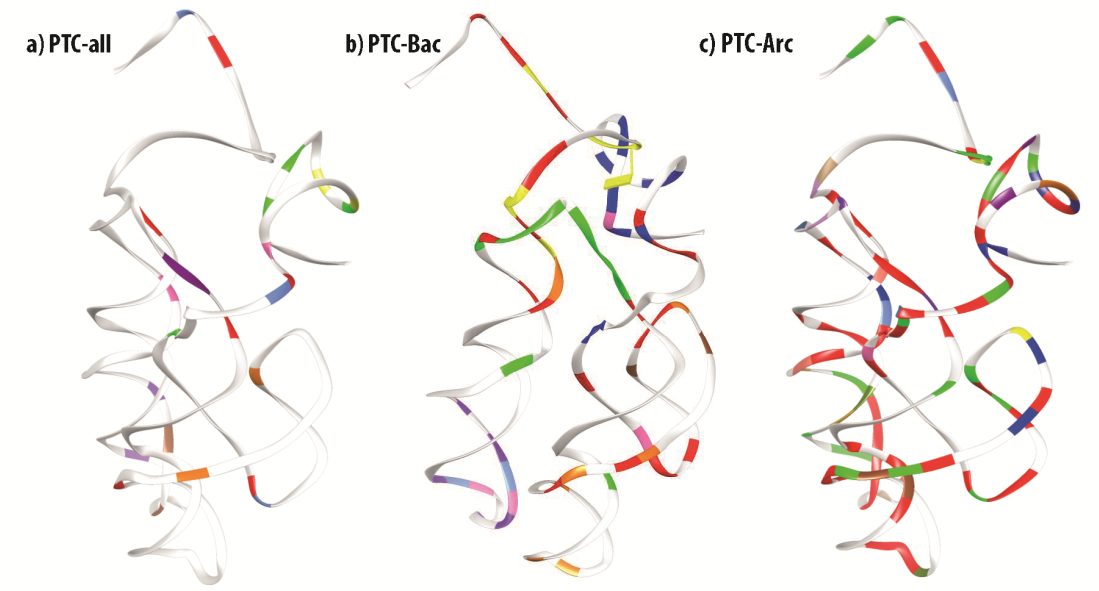


**Figure S3.** Mapping identity elements and information clusters to the 3D structure of (a) PTC-all (b) PTC-Bacteria, and (c) PTC-Archaea.

**Table S1.** Identification of nucleotide positions clustered together by the information analysis on each PTC dataset.

| **Dataset** | **Cluster** | **Nucleotide Positions** |
| --- | --- | --- |
| PTC-all | 1 | 7, 23, 81, 114, 138, 155 |
| PTC-all | 2 | 146, 165, 172, 174 |
| PTC-all | 3 | 5, 137, 154 |
| PTC-all | 4 | 164, 167 |
| PTC-all | 5 | 118, 129 |
| PTC-all | 6 | 78, 79 |
| PTC-all | 7 | 67, 157 |
| PTC-all | 8 | 51, 54 |
| PTC-all | 9 | 42, 56 |
| PTC-Bac | 1 | 5, 7, 9, 18, 19, 23, 27, 67, 71, 81, 114, 117, 127, 135, 137, 138, 142, 154, 155, 157 |
| PTC-Bac | 2 | 63, 70, 72, 73, 74, 76, 77, 78, 79, 80, 94 |
| PTC-Bac | 3 | 145, 146, 156, 164, 165, 167, 171, 172, 174, 176 |
| PTC-Bac | 4 | 8, 11, 12, 13, 20, 21, 22, 24 |
| PTC-Bac | 5 | 99, 104, 110, 115, 118, 126, 129 |
| PTC-Bac | 6 | 42, 48, 51, 54, 56 |
| PTC-Bac | 7 | 139, 175 |
| PTC-Bac | 8 | 124, 130 |
| PTC-Bac | 9 | 44, 52 |
| PTC-Bac | 10 | 43, 53 |
| PTC-Bac | 11 | 25, 26 |
| PTC-Arc | 1 | 7, 11, 22, 23, 30, 32, 33, 45, 51, 52, 53, 54, 55, 63, 65, 69, 78, 79, 81, 91, 93, 95, 101, 102,103, 104, 114, 115, 119, 123, 130, 132, 135, 136, 138, 143, 146, 148, 151, 153, 155, 156,158, 165, 166, 172, 174 |
| PTC-Arc | 2 | 3, 5, 13, 14, 39, 42, 43, 56, 57, 84, 116, 118, 129, 131, 137, 149, 154, 168, 170, 173 |
| PTC-Arc | 3 | 67, 122, 125, 157, 164, 167, 176 |
| PTC-Arc | 4 | 12, 126, 175 |
| PTC-Arc | 5 | 162, 163 |
| PTC-Arc | 6 | 160, 171 |
| PTC-Arc | 7 | 112, 144 |
| PTC-Arc | 8 | 94, 117 |
| PTC-Arc | 9 | 75, 80 |
| PTC-Arc | 10 | 71, 150 |
| PTC-Arc | 11 | 37, 59 |
| PTC-Arc | 12 | 36, 105 |
| PTC-Arc | 13 | 25, 62 |
| PTC-Arc | 14 | 20, 73 |
| PTC-Arc | 15 | 19, 48 |
| PTC-Arc | 16 | 8, 66 |
