# Supplementary material for: The Ancient History of Peptidyl Transferase Center Formation as Told by Conservation and Information Analyses
Source: Life (Basel). 2020 Aug 5;10(8):134. doi: 10.3390/life10080134 (PMC7459865; doi:10.3390/life10080134)
Supplement: Supplementary file 1 [file life-10-00134-s001.zip › life-875402-supplementary/tRNA ancestral sequences.pdf]

tRNA ancestral sequences

>Ala Ancestor

AGCTCATGAGACGACACTTGCACTGCGGAGTGACCGCTA

>Arg ancestor

GTAGCAGCGGGAGCGGCGTTAAATCCGGCCGGCC

>Asn ancestor

GCTGTAGCTGGAAGATTCGGCTGTTAACCGAATGGGTAGGTTCGAATCCTACGGCGA

>Asp ancestor

GAGTTCAGGAGAATACGCCTGTCCGCAGGTCCGGGTTGAGTCCCTCCGGACC

>Cys ancestor

GGGGTATAGCTCAGGAGATTGACTGCAGATCCGGTTCAAATCCGGGTGCCCC

>Gln ancestor

AGCCAGAGCCCGGACTTTGACTCCGGCGTTCGAATCCTGCCATCCCA

>Glu ancestor

CCCGTTCGTAGGGGCACCGCCCTTTCAAGGCGGTGGTGAATCTACGGGAT

>Gly ancestor

CAGATAAGTTCAGACATAACTTGCCAAGGTCGTTCGATTCGTTCTGC

>His ancestor

GCCGCGGTAGTGTAGCACATCAGATTGTGGCCCGGGTTCGAATCCCGGCCGCGGC

>Ile ancestor

GCCGCTCAGGAACTCGGGCTAATACGGGTTCGACCCCCT

>Leu ancestor

GGCGAGGCCGGATTAAGCGTGGTCGATCCCACCGCTGCC

>Lys ancestor

GGGCCCGTAGCTCGGCAGCGCTTGGCTTTTACCGG

>Met ancestor

GTGCTCGGGCCGGCTCATAACCGGGAGTGCGGTCGAATCCCGCCGGCGCC

>Phe ancestor

GGTAGTCAGGGAGCGCCGGACTGAAGCCGGTTCAAATCCGGGCCGCGG

>Pro ancestor

GGGTGTAGCGCAGGCACCTGCTTTGGGAGGGGGGCGTGGTTCAAATCCCGCCACCCCG

>Sel ancestor

GGGCGCCCGGTTTCAAAACCGGTGGTCCAGTGGTTCGACTCCCCT

>Ser ancestor

GGCTGGACTGAACGAATCCCCGACA

>Thr ancestor

AGTGGTGGGCTGGGCCGTACCAGATGGATCGAAGCCATCT

>Trp ancestor

CGTGGCTGAGCCGGGCTCCAGTTGGGTTCAAATCCCACCGGCCCC

>Tyr ancestor

CGGTAGCTCAGGGGACTGTAGGTGGGGGTTCGAATC

>Val ancestor

GTTTAGGATCACGTCCCTAACAGCAGAAGGTCCTTGAAGGGTGAACC
