# Supplementary figures and images for: The Ancient History of Peptidyl Transferase Center Formation as Told by Conservation and Information Analyses
Source: Life (Basel). 2020 Aug 5;10(8):134. doi: 10.3390/life10080134 (PMC7459865; doi:10.3390/life10080134)

**a) PTC\_all**

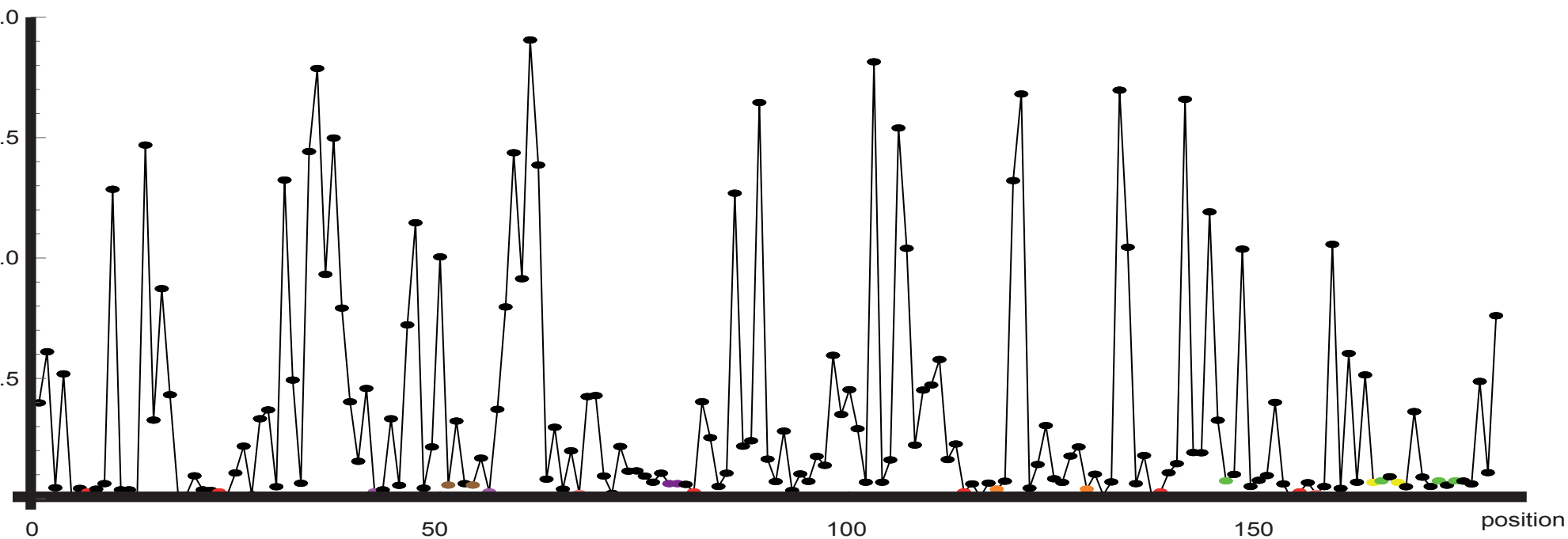

**b) PTC\_Bacteria**

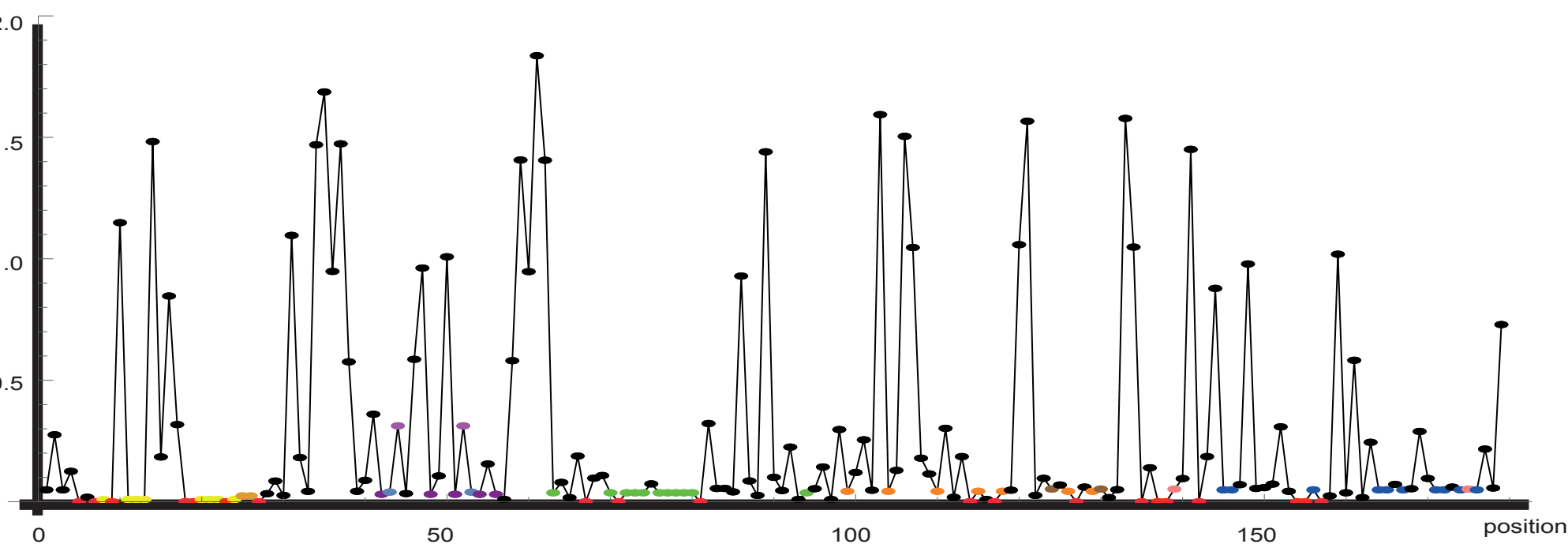

**c) PTC\_Archaea**

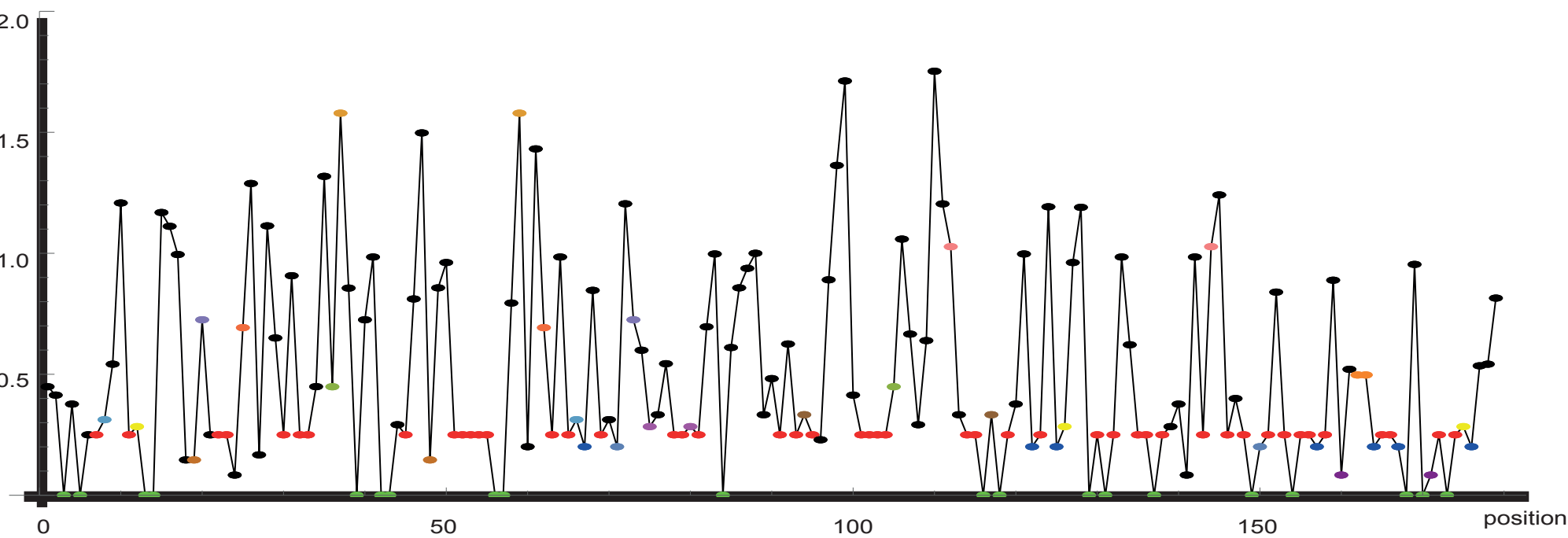

Supplement: Supplementary file 1 [file life-10-00134-s001.zip › life-875402-supplementary/Figure S1.pdf]

a)

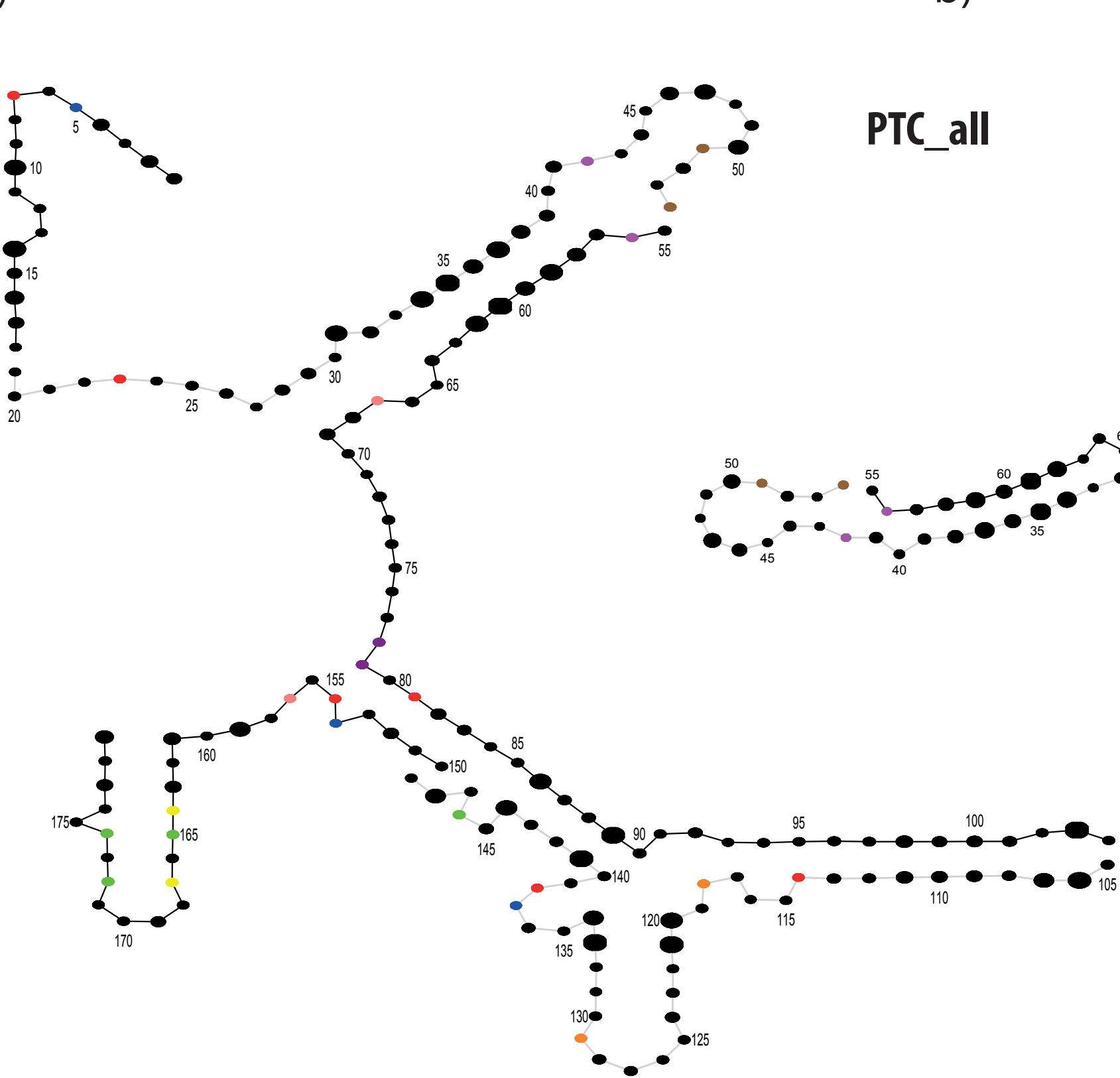

b)

PTC\_all

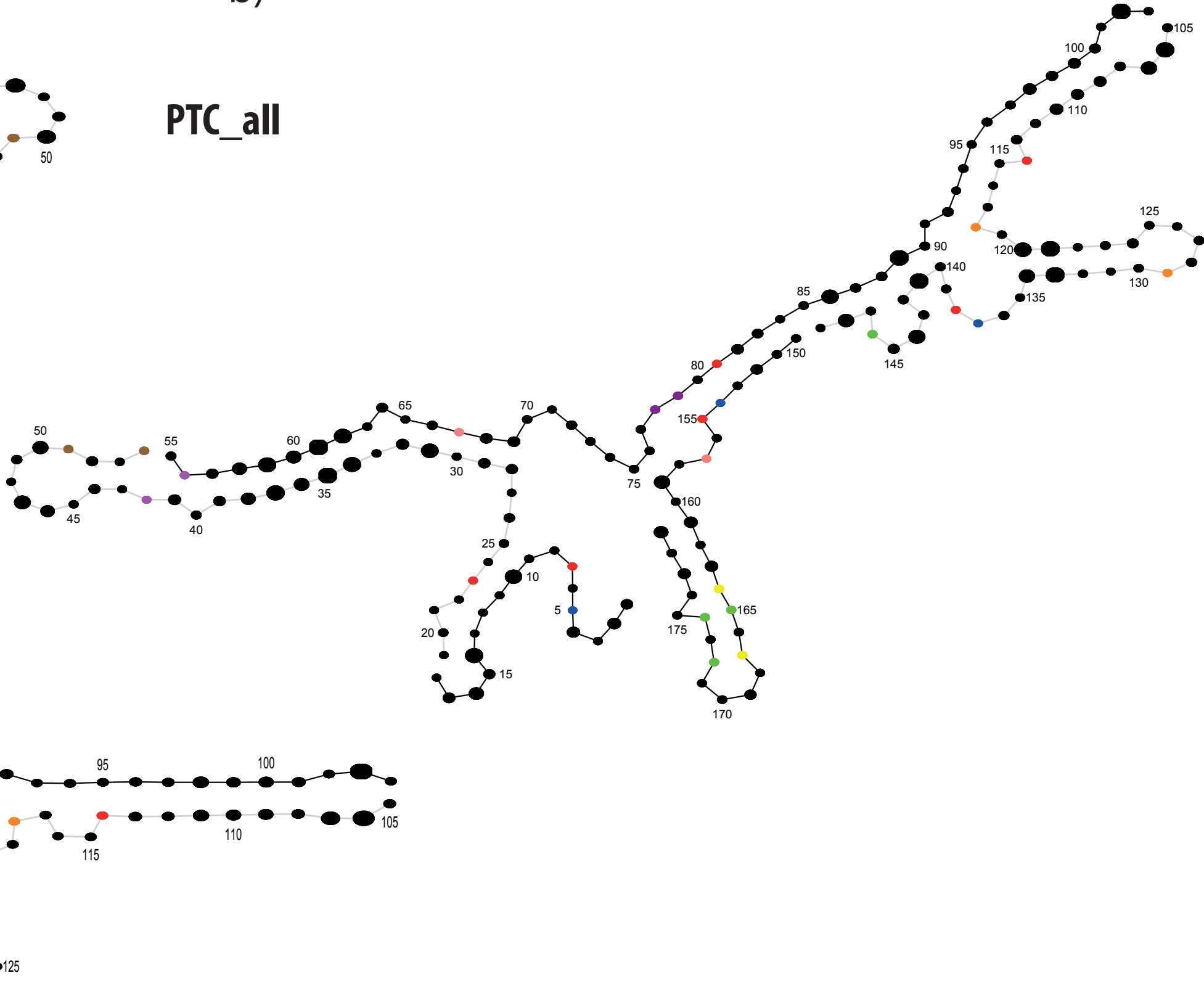

c)

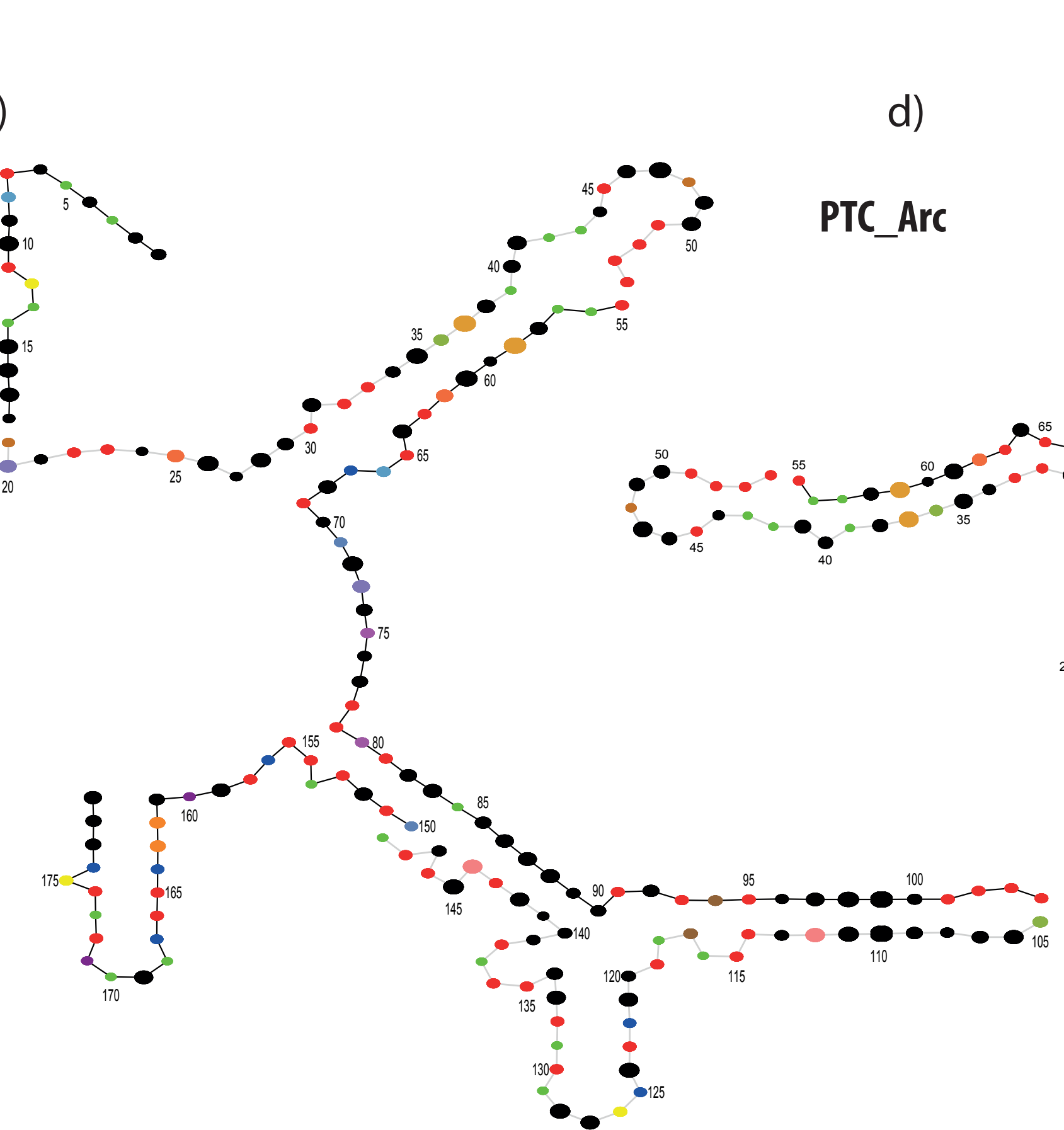

d)

PTC\_Arc

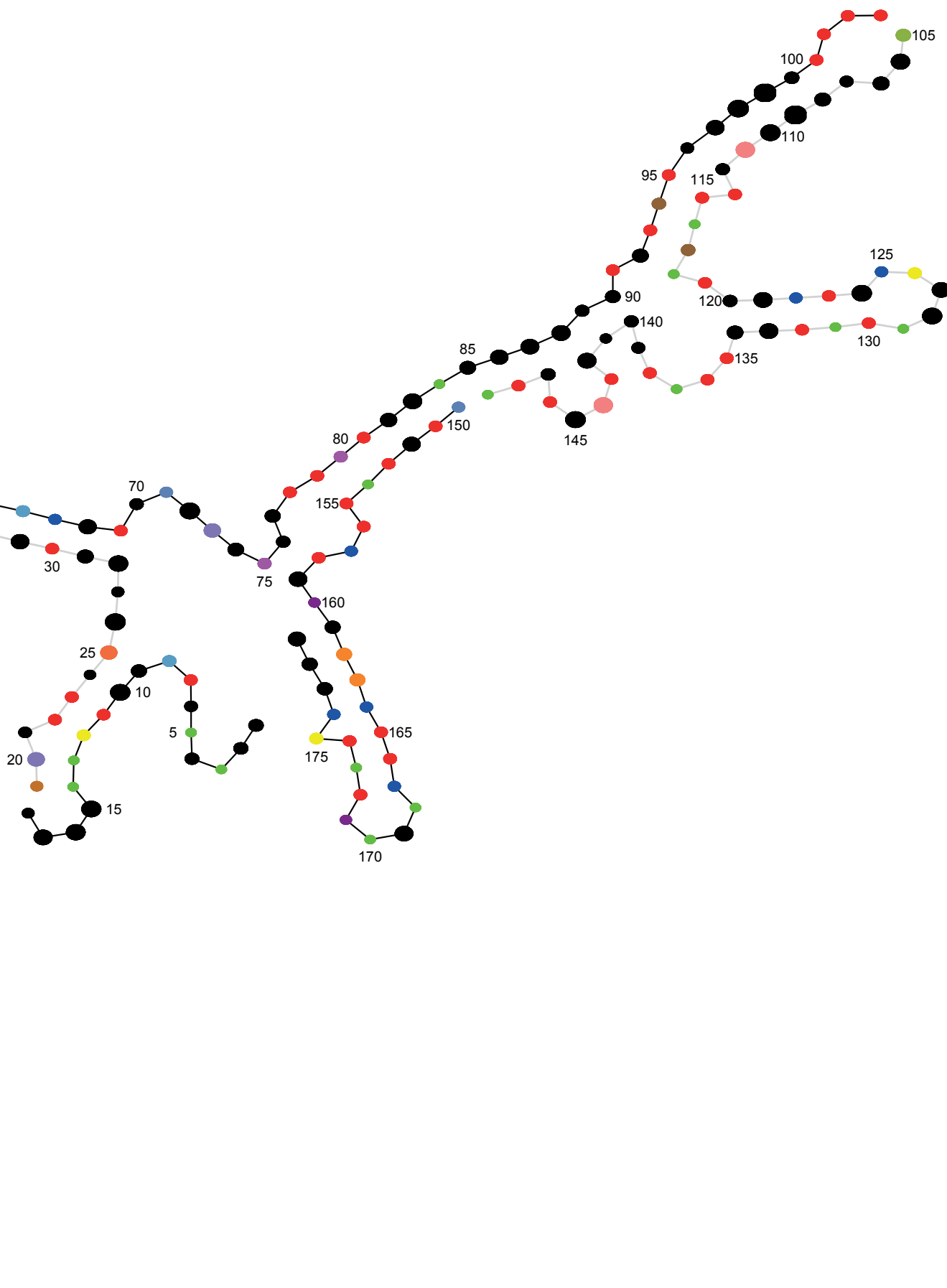

Supplement: Supplementary file 1 [file life-10-00134-s001.zip › life-875402-supplementary/Figure S2.pdf]

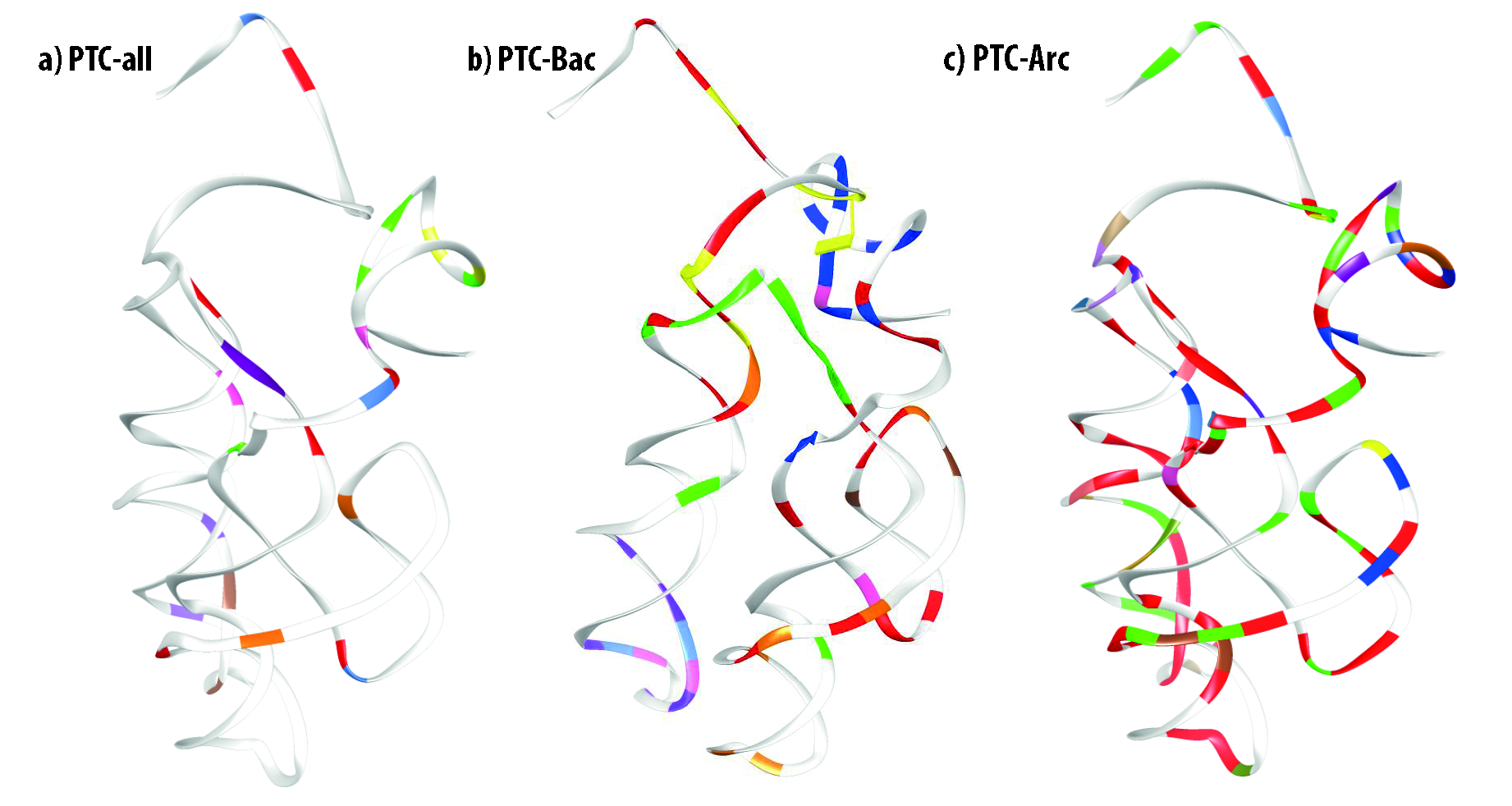

Supplement: Supplementary file 1 [file life-10-00134-s001.zip › life-875402-supplementary/Figure S3.tif]
